# Ultrasound-Based Therapies in Primary Central Nervous System Tumors

**DOI:** 10.3390/cancers18061010

**Published:** 2026-03-20

**Authors:** Giovanni Dima, Alessandro Olivari, Vincenzo Di Nunno, Marta Aprile, Alicia Tosoni, Lidia Gatto, Chiara Maria Argento, Marzia Margotti, Stefania Bartolini, Alfredo Conti, Enrico Franceschi

**Affiliations:** 1 Nervous System Medical Oncology Department, Istituto di Ricovero e Cura a Carattere Scientifico (IRCCS) Istituto Delle Scienze Neurologiche di Bologna, Bellaria Hospital, 40139 Bologna, Italy; 2 Medical Oncology Unit 2, Santa-Chiara Hospital, 56126 Pisa, Italy; 3 Medical Oncology Unit, Parma University Hospital, 43125 Parma, Italy; 4 Department of Neurosurgery, Istituto di Ricovero e Cura a Carattere Scientifico (IRCCS) Istituto Delle Scienze Neurologiche di Bologna, 40139 Bologna, Italy; 5 Dipartimento di Scienze Biomediche e Neuromotorie (DIBINEM), Alma Mater Studiorum Università di Bologna, 40126 Bologna, Italy

**Keywords:** CNS tumors, focused ultrasound, sonodynamic therapy, blood–brain barrier, reactive oxygen species, nanotheranostics, AI-guided therapy, multimodal neuro-oncology

## Abstract

Primary central nervous system (CNS) tumors remain a major challenge in neuro-oncology due to their cellular heterogeneity, infiltrative growth, and the protective blood–brain barrier (BBB), which limits the effectiveness of systemic therapies. Despite aggressive multimodal treatments, patient survival remains poor, highlighting the urgent need for new therapeutic strategies. Ultrasound-based therapies, including focused ultrasound (FUS) and sonodynamic therapy (SDT), have emerged as promising approaches. FUS can transiently open the BBB and induce localized mechanical and thermal effects, enhancing drug delivery, while SDT activates tumor-specific sensitizers to generate reactive oxygen species that trigger cancer cell death. Preclinical and early clinical studies suggest that combining these modalities with chemotherapy, immunotherapy, or radiotherapy may improve treatment outcomes. Emerging tools such as AI-guided monitoring, theranostic platforms, and ultrasound-responsive nanoparticles could further enable personalized interventions. However, challenges remain, including protocol variability, tumor heterogeneity, and limited long-term safety data. Careful optimization and clinical validation are needed before these strategies can be widely adopted in the management of CNS tumors.

## Simple Summary

Primary central nervous system tumors are difficult to treat due to their complex biology and the protective blood–brain barrier, which limits drug delivery. Ultrasound-based therapies are emerging as promising non-invasive strategies to overcome these challenges. Focused ultrasound can temporarily open the blood–brain barrier and enhance drug delivery, while sonodynamic therapy can selectively kill tumor cells through the generation of reactive oxygen species. These approaches may improve the effectiveness of existing treatments such as chemotherapy and immunotherapy. Although early results are encouraging, further research is needed to optimize treatment protocols and confirm long-term safety and efficacy.

## 1. Introduction

Primary central nervous system (CNS) tumors continue to pose major therapeutic and biological challenges in neuro-oncology, particularly due to their intrinsic heterogeneity and limited treatment responsiveness. Their complex biology, heterogeneous cellular composition, and the protective environment of the brain make effective treatment particularly difficult. In malignant CNS tumors, systemic therapies often fail to reach therapeutic concentrations due to the BBB, which tightly regulates substance passage into the brain [1]. Despite an aggressive multimodal strategy, median survival for patients with high-grade tumors such as glioblastoma remains poor [2]. Notably, these outcomes have shown only marginal improvement over the past decades, underscoring the urgent need for innovative therapeutic strategies capable of overcoming biological and anatomical treatment barriers. Despite decades of research and therapeutic advances, survival outcomes have improved far less than we would have hoped. This makes it increasingly clear that new strategies are needed to address the biological and anatomical barriers that continue to limit treatment efficacy. In everyday clinical practice, it is evident that not all tumor regions visible on FLAIR imaging are equally vascularized. Some areas remain poorly perfused, and this alone can severely limit the access of systemically administered drugs to infiltrative and peritumoral tissue. As a consequence, meaningful portions of the tumor may never be fully exposed to therapy, even when patients receive intensive systemic regimens. In this setting, ultrasound-based approaches are attracting growing attention as a promising adjunct to conventional systemic treatments. By transiently modulating the BBB, ultrasound can increase intratumoral drug concentrations while simultaneously enhancing tumor sensitization through combination with chemotherapy, immunotherapy, or radiotherapy. This approach could potentially overcome multidrug resistance, reduce systemic toxicity by enabling lower drug doses, and improve overall treatment outcomes. [3] However, the extent to which these theoretical advantages translate into consistent clinical benefit remains an open question. Two main ultrasound modalities have been explored in this context: SDT and FUS. SDT employs low-intensity ultrasound to activate sonosensitizers that accumulate in tumor tissue, producing reactive oxygen species (ROS) that induce apoptosis and necrosis [4,5]. FUS, meanwhile, exerts localized mechanical and thermal effects and can transiently open the BBB, facilitating drug delivery directly to tumor regions. A recent review by Alrashidi et al. looked at the use of focused ultrasound in glioblastoma from a mainly clinical and translational perspective [6]. Their work gives a useful snapshot of where the field currently stands. What it does not really cover, however, are the underlying physical mechanisms—how ultrasound actually interacts with cells, what happens at the microscale, and why certain biological effects emerge. In this review, we focus precisely on those aspects. The biophysical principles are examined, along with the behavior of cells in vitro under sonication conditions and the acoustic processes that drive these responses. In this way, the two reviews complement each other, but they address different levels of the problem [6]. This review provides a comprehensive overview of current ultrasound-based therapies for primary CNS tumors, focusing on both preclinical and clinical evidence. We discuss the underlying mechanisms, evaluate the combination of ultrasound with systemic therapies, address current challenges, and outline future directions. By highlighting recent advances and ongoing research, this review aims to offer a clear perspective on how ultrasound-based approaches could reshape treatment paradigms for CNS tumors.

## 2. Mechanisms of Ultrasound-Based Therapies in CNS Tumors

Ultrasound-based therapies act through multiple, partially overlapping physical and biological mechanisms, which together enable targeted intervention in primary CNS tumors. These mechanisms include thermal ablation, mechanical disruption via cavitation phenomena, and modulation of the BBB, collectively enabling precise tumor targeting while minimizing damage to surrounding healthy tissue. Focused ultrasound is not intrinsically a thermal treatment. Whether heat becomes relevant or not depends on how the energy is delivered. At high intensities, temperature rises can be sufficient to damage proteins and membranes, leading to coagulative necrosis. At lower intensities, instead, temperature changes are modest, and the biological effects arise mainly from mechanical forces and cavitation. In these cases, cells are not ‘burned’ but physically stressed by the acoustic field. This thermal ablation effect is spatially confined due to the focused nature of the ultrasound beam, allowing for non-invasive tumor debulking with high precision. Moreover, thermal effects can trigger vascular damage and ischemia, exacerbating tumor cell death. However, in CNS tumors, especially gliomas, complete thermal ablation is challenging due to tumor heterogeneity and infiltrative growth patterns, necessitating complementary mechanisms to enhance efficacy [7,8,9]. It should be noted that primary ablative approaches using HIFU ablation, histotripsy and MB-FUS represent an active and interesting research area in neuro-oncology; briefly, histotripsy mechanically fractionates tissue via controlled acoustic cavitation, HIFU induces thermal coagulative necrosis through concentrated acoustic energy deposition, and MB-FUS transiently disrupts the blood–brain barrier via intravenous microbubbles to enhance locoregional drug delivery [10,11,12,13].

In this context, incomplete ablation and tumor infiltration beyond the sonicated volume represent clinically relevant limitations. Mechanical effects, primarily mediated by cavitation, significantly contribute to tumor destruction. Cavitation involves oscillation of microbubbles—either endogenous gas pockets or exogenously administered contrast agents—under ultrasound exposure. Stable cavitation induces microstreaming and shear stress on cellular membranes, whereas inertial cavitation causes bubble collapse, generating shock waves and microjets that physically disrupt tumor cells and vasculature.

Over the past few years, it has become increasingly obvious that microbubbles are not all the same and that these differences matter a great deal when we use ultrasound in biological systems. Features that might once have seemed like minor technical details—such as how big the bubbles are, what their shell is made of, or how stiff or flexible they are—actually change the way they behave under sound waves. A recent study by Martinez et al. illustrates this very clearly. They compared microbubbles of different sizes and compositions and showed that each type produces its own acoustic ‘signature,’ which in turn shapes how strongly the blood–brain barrier opens and how much sterile inflammation is triggered [14]. Larger, more compliant bubbles tended to generate richer acoustic activity and a stronger inflammatory response, while smaller or stiffer ones led to more subdued effects. Of particular note is the observation that even the pattern of sonication—how many spots are targeted and in what order—can amplify or soften these responses. Although their work focuses on BBB opening, the broader message applies widely: the choice of microbubble is not a trivial decision. It actively shapes the biology we observe, and treating it as an experimental variable rather than a fixed reagent can completely change the outcome of a sonication experiment. These forces not only cause direct cytotoxicity but also modulate the tumor microenvironment by enhancing immune cell infiltration and promoting pro-inflammatory signaling cascades. Cavitation-mediated mechanical disruption further enhances tumor vasculature permeability, facilitating improved therapeutic agent penetration [15]. A critical advancement is the transient, reversible opening of the BBB induced by low-intensity focused ultrasound (LIFU) in the presence of circulating microbubbles. The BBB is a major obstacle to effective systemic therapy due to its tight junctions and selective permeability. LIFU-mediated BBB disruption mechanically stresses endothelial cells, inducing reversible loosening of tight junctions, increased transcytosis, and enhanced paracellular permeability in a highly localized, MRI-guided manner. This transient modulation overcomes a major therapeutic barrier by enabling effective penetration of systemic drugs into tumor and peritumoral regions, thereby improving drug bioavailability and efficacy without significantly compromising overall BBB integrity [5,16,17,18]. Nevertheless, inter-patient variability in vascular anatomy and microbubble dynamics can substantially influence the reproducibility of BBB opening. Preclinical studies have shown that ultrasound-mediated BBB opening significantly enhances the delivery and efficacy of chemotherapeutics such as temozolomide, carboplatin, and carmustine, as well as monoclonal antibodies and nanoparticle-based therapies [19]. This method preserves BBB integrity within hours post-treatment, minimizing risks of neurotoxicity and infection. These molecules are usually described as photosensitizers, but this definition is actually incomplete. In practice, many of them respond not only to light but also to ultrasound. This happens because ultrasound does not simply “shake” the tissue: through cavitation and rapid pressure oscillations, it creates highly localized and transient physical conditions that are able to transfer energy to the sensitizer molecules. In this sense, ultrasound provides an alternative way to excite these compounds, even in the absence of photons [20,21].

The result is that the same molecules that generate ROS under light irradiation can also do so under ultrasound exposure. This dual behavior is what makes sonodynamic therapy fundamentally different from photodynamic therapy and explains why SDT can be applied in deep tissues where light cannot reach, while still preserving molecular selectivity. Compared to photodynamic therapy, SDT benefits from deeper tissue penetration of ultrasound waves, allowing treatment of deep-seated CNS tumor [22]. Importantly, adequate tissue oxygenation remains a prerequisite for effective ROS generation, potentially limiting SDT efficacy in hypoxic tumor regions [23]. Beyond direct cytotoxicity, SDT modulates the tumor microenvironment by promoting immunogenic cell death characterized by the release of damage-associated molecular patterns (DAMPs), increased antigen presentation, and recruitment of cytotoxic T lymphocytes. These immunomodulatory effects offer promising opportunities for combination with immunotherapies, such as immune checkpoint inhibitors, potentially overcoming glioma-associated immunosuppression and improving clinical outcomes [24]. The combination of LIFU-mediated BBB opening and SDT presents significant potential for synergistic integration with systemic therapies. LIFU facilitates increased penetration of chemotherapeutic, targeted, and immunotherapeutic [25,26] agents, thereby augmenting their intratumoral concentration and efficacy. Concurrently, SDT-induced oxidative stress and immunogenicity may sensitize tumors to immunotherapies by overcoming immune evasion mechanisms typical of gliomas and other primary CNS tumors. Taken together, this dual strategy combines improved drug delivery with immune modulation, potentially enabling a more comprehensive therapeutic impact on tumor cells [27]. Whether this synergistic effect can be sustained across multiple treatment cycles in a clinical setting has yet to be determined.

**Figure 1:**
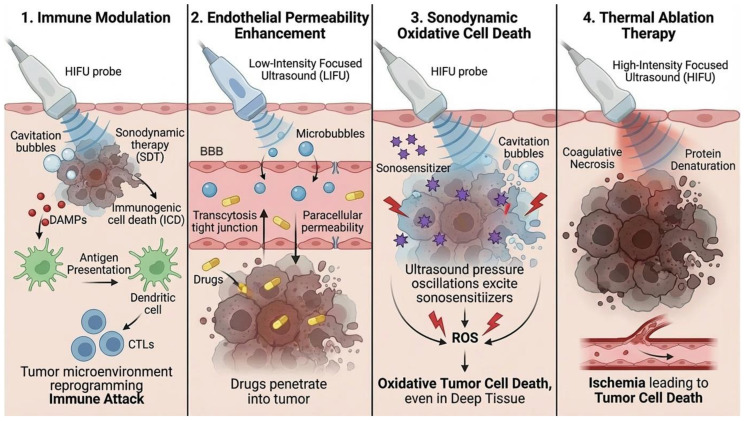
Schematic illustration of the combined effects of SDT and FUS in CNS tumors. The figure depicts the synergistic interaction between FUS-mediated BBB opening, which enhances drug and sonosensitizer delivery to tumor cells, and SDT activation, which generates ROS leading to apoptosis. The combined effect results in enhanced cytotoxicity and improved immunotherapy responsiveness. Current research focuses on optimizing ultrasound parameters—including intensity, frequency, and duty cycle—alongside sonosensitizer pharmacodynamics and treatment timing to maximize therapeutic synergy while minimizing off-target effects. Preclinical glioma models have demonstrated improved tumor control and prolonged survival with these combined modalities compared to monotherapies. These integrative strategies hold promise to redefine multimodal treatment paradigms for primary CNS tumors and improve clinical outcomes in these challenging malignancies [28]. Consistent with this mechanistic framework, the AJNR review by Nabavizadeh et al. [29] delineates how focused ultrasound integrates biophysical effects, MRI-guided targeting and BBB modulation to bridge preclinical innovation and early-phase clinical translation in primary brain tumors.

The mechanisms and characteristics of SDT and FUS are compared in [Table 1] and illustrated in [Figure 1].

To contextualize the energy levels involved in therapeutic ultrasound, it is useful to compare them with those used in conventional diagnostic ultrasound. Diagnostic ultrasound, including intraoperative neurosonography used during brain surgery, operates at spatial-peak temporal-average intensities (ISPTA) typically below 720 mW/cm^2^ with no known adverse biological effects [30]. In contrast, therapeutic FUS-mediated BBB opening uses low-intensity pulsed protocols with ISPTA values ranging from approximately 0.1 to several W/cm^2^, while high-intensity focused ultrasound (HIFU) for ablation can reach values exceeding 1000 W/cm^2^ at the focal point [6,31].

This represents energy delivery several orders of magnitude greater than diagnostic modalities, underscoring the need for careful parameter control and real-time monitoring in therapeutic settings.

## 3. Application of Ultrasound Modalities in Primary CNS Tumors

Therapeutically, SDT uses ultrasound to activate sensitizing agents such as 5-ALA, selectively inducing cytotoxic effects in tumor cells; recent clinical investigations in recurrent glioblastoma patients reported favorable safety profiles and preliminary efficacy, with a median survival of 14 months, compared to 12 months for patients treated with conventional therapies. These results suggest SDT as a promising non-invasive treatment for otherwise inoperable or resistant tumors [32]. Concurrent studies on LIPU have shown its ability to transiently open the BBB, significantly enhancing the delivery of chemotherapeutics like carboplatin and paclitaxel to gliomas. Phase I/II clinical trials confirmed effective BBB disruption in the majority of treatment sessions, with improved drug penetration correlating with prolonged progression-free survival (PFS) of 7.2 months in treated patients, compared to 4.5 months in controls. These findings, while encouraging, should be interpreted cautiously given the limited sample sizes and non-randomized study designs. Multicenter studies further supported these findings, demonstrating variable but encouraging local tumor control and therapeutic response with combined ultrasound and chemotherapy regimens. However, optimization of sonication parameters remains essential to maximize efficacy while minimizing adverse effects [33]. Beyond BBB modulation, thermo-sonic ablation using pulsed ultrasound waves has shown increased tumor necrosis in preclinical models, suggesting a role for focused ultrasound in localized tumor destruction with potentially reduced collateral damage [34]. Moreover, surgical outcomes have benefited from the integration of sonication technologies; combining focused ultrasound with hyperspectral imaging enhances tumor margin delineation during resection, potentially reducing residual disease and recurrence, while adjunctive tools such as CyberKnife radiosurgery and intraoperative magnetic resonance imaging (MRI) complement sonication to optimize tumor removal and preserve healthy tissue [35]. Recent clinical and preclinical studies summarized in Table 2, have demonstrated that ultrasound-mediated BBB opening—using FUS, LIFU, microbubbles, or implantable devices—or SDT can be combined with chemotherapy, radiotherapy, immunotherapy, and diagnostic techniques to enhance treatment efficacy in primary CNS tumors. For instance, a phase I/II clinical trial using a nine-emitter implantable ultrasound device (SonoCloud-9) activated every three weeks in patients with recurrent glioblastoma treated with carboplatin confirmed that repeated BBB disruption is safe, well-tolerated, and associated with measurable pharmacokinetic effects and BBB opening on imaging. This trial also reported a median survival of 12.5 months, compared to 10 months in patients receiving standard chemotherapy without BBB disruption [36]. Another trial (LIBERATE) is investigating whether MRI-guided LIFU combined with microbubbles can improve the sensitivity of liquid biopsy in glioblastoma patients by increasing circulating free and tumor DNA in blood samples, potentially facilitating personalized systemic therapies [37]. In pediatric diffuse intrinsic pontine glioma (DIPG), preclinical studies showed that FUS-mediated BBB opening significantly increased the intratumoral concentration of panobinostat, resulting in a 40% reduction in tumor volume and prolonged survival in murine models [38]. Moreover, the combination of FUS-mediated BBB opening with moderately hypofractionated radiotherapy (39 Gy) in brainstem diffuse midline glioma demonstrated feasibility and safety without significant additional toxicity. Importantly, preliminary evidence suggests that BBB opening via FUS can modulate the tumor microenvironment, including lymphocyte infiltration, which could have immunotherapeutic implications [39].

Table 2 comprehensive overview of diagnostic and therapeutic applications of ultrasound technologies in primary CNS tumors, including FUS, low-intensity pulsed ultrasound (LIPU), SDT, and ultrasound-enhanced liquid biopsy (“sonobiopsy”). Both clinical and preclinical studies are included, and detailed technical parameters such as ultrasound device type, emission frequency, and microbubble [43] formulation are reported to enhance reproducibility across studies. The table highlights key translational advances such as implantable ultrasound systems (e.g., SonoCloud-9), MRI-guided FUS platforms, and the integration of ultrasound with chemotherapy, radiotherapy, and immunotherapy.

### Recent Updates

In 2025, new clinical and technological evidence further strengthened the therapeutic potential of ultrasound-based modalities in primary CNS tumors. Preliminary clinical results from Alpheus Medical reported the first-in-human application of sonodynamic therapy (SDT) in newly diagnosed glioblastoma, showing a favorable safety profile and early signs of efficacy, with median survival improvement compared to historical controls, although data remain limited to early-phase, non-randomized studies [44]. Concurrently, ongoing multicenter trials—such as SONOCLOUD-9 (phase I-II trial) and SONOBIRD (NCT05902169 a randomized phase 3 trial)—continued to evaluate implantable and microbubble-based systems for repeated and controlled BBB modulation, with interim results of the former presented at major international neuro-oncology conferences [36], and subsequently published in full while the latter is currently enrolling, and no interim data are yet available.

On the technological side introduced the concept of Acoustic Emission Dose (AE-dose) as a quantitative parameter to standardize the intensity and safety of FUS-mediated BBB opening, thereby reducing inter-patient variability [45]. Moreover, a recent systematic review summarized ongoing and completed focused ultrasound (FUS) trials for brain tumors, highlighting a gradual transition from feasibility studies to efficacy-driven clinical testing [44]. Despite these encouraging developments, the clinical translation of ultrasound-based therapies still faces substantial challenges related to standardization, safety monitoring, and long-term validation—issues that continue to define the next frontier of research in neuro-oncologic ultrasound applications.

## 4. Limitations and Future Challenges

Despite promising advances, ultrasound-based therapies—particularly FUS and SDT—face several limitations that hinder broader clinical translation.

### 4.1. Variability and Reproducibility of Treatment Parameters

Ultrasound treatment parameters—including acoustic frequency, peak negative pressure, pulse duration, duty cycle, microbubble concentration, and sonosensitizer dose—show substantial variability, affecting both safety and therapeutic outcomes. [5,46] Phase 0 clinical studies in glioma patients have highlighted the need for patient-specific calibration, as cavitation thresholds vary significantly with anatomical and vascular differences. The effectiveness of SDT also depends on pharmacokinetics, tumor-selective accumulation of sonosensitizers, and tissue oxygenation, all of which influence ROS generation and apoptotic response [47,48].

Even though the lack of standardized protocols for ultrasound parameters represents a focal point to consider for cross-study comparisons [37], this heterogeneity is currently expected and appropriate, given that multiple concepts and variables within each concept are actively being explored, such as sonosensitizer type and dosing, microbubble administration, and imaging guidance. Harmonization through established frameworks, such as IDEAL or CONSORT, could improve reproducibility and facilitate wider adoption [48].

### 4.2. Tumor Heterogeneity and Blood–Brain Barrier Variability

Gliomas are highly heterogeneous, exhibiting irregular vasculature, hypoxic niches, and infiltrative margins, which affect both the extent and uniformity of FUS-mediated BBB opening and intratumoral drug distribution. Distinguishing between the BBB and the blood-tumor barrier (BTB) adds complexity: while the BTB is often more permeable, it is less predictable, and tumor margins frequently retain an intact BBB, potentially shielding infiltrating tumor cells from therapy [49].

This heterogeneity also limits the reproducibility of SDT, as adequate tissue oxygenation [50] is essential for ROS generation, and pharmacokinetic variability [47] affects sonosensitizer accumulation.

### 4.3. Safety Concerns and Monitoring Limitations

FUS-mediated BBB disruption has generally been safe in early-phase trials, yet adverse events—including microhemorrhages, vasogenic edema, sterile inflammation, and microvascular or parenchymal injury—have been reported, particularly with high acoustic pressures or improper microbubble dosing [38,51,52,53,54]. Monitoring tools, such as real-time cavitation mapping, MRI thermometry, and acoustic emission detection, can mitigate these risks but are not universally available, standardized, or technically straightforward, especially in pediatric patients or deep-seated lesions [39,52,55]. Repeated BBB opening raises questions about long-term neurotoxicity, immune activation, inflammation, and potential cognitive or structural consequences, which remain poorly characterized [56]. Off-target and systemic effects—such as unintended tissue heating or sonosensitizer accumulation in extracerebral organs—are uncommon but should be considered [57].

### 4.4. Limited Long-Term Clinical Data

Most clinical studies remain early-phase (Phase 0–II), primarily assessing feasibility and safety rather than long-term outcomes such as PFS or overall survival [36,58]. The lack of standardized sonication protocols, imaging criteria, and reporting metrics hampers inter-study comparisons and meta-analyses [59]. Notably, the ongoing phase 3 registration trial NCT05902169 (SONOBIRD) is currently evaluating this approach in patients with progressive GBM IDHwt, which is expected to provide higher-level evidence on long-term efficacy and safety.

### 4.5. Pediatric Applications and Ethical Considerations

In pediatric patients, including those with diffuse midline gliomas (e.g., DIPG), FUS therapy presents unique challenges due to skull thickness, ongoing brain development, and long-term safety concerns. Repeated BBB disruption in the developing brain raises ethical questions. Ongoing myelination, synaptogenesis, and vascular plasticity increase susceptibility to unintended structural or cognitive effects, highlighting the need for longitudinal monitoring and pediatric-specific safety thresholds [60].

## 5. AI and Theranostics

Recent technological advances have positioned artificial intelligence (AI), theranostic systems, and nanotechnology as pivotal enablers for next-generation ultrasound-based therapies in primary CNS tumors. The integration of these tools aims to enhance precision, real-time monitoring, and personalization of treatment while maintaining safety and reproducibility across patients. AI-based image analysis and signal processing are increasingly employed for real-time cavitation monitoring and treatment optimization during FUS procedures. Machine learning algorithms can automatically classify acoustic emissions to distinguish between stable and inertial cavitation, allowing clinicians to dynamically adjust acoustic power and duty cycles to ensure controlled BBB opening without tissue damage [61]. Deep learning–driven MRI segmentation further facilitates accurate tumor delineation, intraoperative targeting, and post-sonication assessment, minimizing inter-operator variability. These developments support the transition toward closed-loop FUS systems, in which AI-driven feedback autonomously modulates ultrasound parameters according to patient-specific anatomical and physiological responses [62]. Parallel to AI integration, theranostic platforms are reshaping the conceptual framework of ultrasound-based neuro-oncology. Theranostics merges diagnostic imaging and therapeutic delivery into a unified workflow, leveraging contrast-enhanced ultrasound, MRI, or PET for real-time visualization of BBB permeability and therapeutic distribution. MRI-guided FUS systems now enable simultaneous drug delivery and intraprocedural monitoring, while microbubble- or nanoparticle-based contrast agents serve dual diagnostic and therapeutic functions, enhancing treatment precision. For instance, liposomal or polymeric nanoparticles loaded with chemotherapeutics or sonosensitizers can be activated by ultrasound to achieve spatiotemporal control of drug release and local cytotoxicity [63].

## 6. Smart Nanotechnology and Nanocarriers

Smart nanoparticles, specifically ultrasound-responsive nanocarriers, have emerged as a cornerstone of this paradigm. Engineered systems such as perfluorocarbon-based nanodroplets, porphyrin–lipid nanovesicles, and polymeric micelles exhibit phase-transition or ROS-responsive behaviors upon sonication, enabling both mechanical and chemical enhancement of therapy. These nanosystems can be functionalized with ligands targeting tumor-specific biomarkers such as EGFRvIII or integrins, ensuring selective accumulation in glioma tissue and reducing off-target toxicity [64,65]. Moreover, combining ultrasound-triggered release with immunotherapeutic payloads—such as siRNA, checkpoint inhibitors, or cytokine-encoding plasmids—may potentiate immune activation in the tumor microenvironment, an area currently under preclinical investigation [66].

An important and currently underexplored question concerns the physical effects that ultrasound-based treatments may exert on nanostructures and the extracellular matrix (ECM). While much attention has been paid to the biological consequences of sonication, it remains unclear how ultrasound energy interacts with nanotubes and other nanocarriers at the structural level. Acoustic forces, cavitation-induced shear stress, and thermal microeffects could potentially alter the integrity, drug-loading capacity, or surface functionalization of nanotubes, with direct implications for drug release kinetics and therapeutic efficacy [67]. Similarly, the ECM—a key determinant of tumor stiffness, drug diffusion, and immune cell infiltration—may be remodeled by repeated sonication in ways that are not yet fully characterized. These uncertainties warrant dedicated preclinical investigation before clinical translation of nanotube-based ultrasound strategies can proceed with confidence.

Overall, the integration of AI-assisted focused ultrasound, ultrasound-responsive theranostic systems, and image-guided approaches may represent an important step toward more personalized and adaptive strategies in neuro-oncologic treatment, although clinical validation remains necessary. As these technologies mature, they promise to overcome long-standing barriers in CNS drug delivery, safety monitoring, and therapeutic selectivity, paving the way for the clinical realization of intelligent ultrasound-guided cancer therapy.

## 7. Conclusions and Future Outlook

Ultrasound-based therapies represent a rapidly evolving frontier in neuro-oncology, offering a potentially effective treatment option for CNS tumors. Through mechanisms such as transient BBB modulation, selective sonodynamic activation, and immune microenvironment reprogramming, both FUS and SDT have demonstrated the ability to enhance therapeutic delivery and antitumor efficacy while minimizing systemic toxicity.

Since these are emerging techniques, there are significant limitations to their inclusion in clinical practice. These include the lack of standardized acoustic dosimetry, variability in BBB response, limited long-term safety data, and uncertainties regarding optimal treatment sequencing and patient selection. Furthermore, the biological complexity of gliomas—marked by intratumoral heterogeneity, hypoxia, and an immunosuppressive microenvironment—continues to challenge the reproducibility and durability of ultrasound-mediated therapeutic effects. Adverse effects such as microhemorrhage, vasogenic edema, and neuroinflammatory changes, although generally reversible, underscore the necessity for stringent procedural monitoring and risk stratification. Looking ahead, the convergence of artificial intelligence, real-time cavitation analytics, and ultrasound-responsive nanotechnology promises to transform these limitations into actionable opportunities for adaptive and personalized therapy.

To fully realize this potential, future research must prioritize large-scale, randomized, multicenter clinical trials to establish definitive evidence of efficacy, safety, and cost-effectiveness. Standardization of ultrasound parameters, harmonization of safety reporting, and long-term follow-up will be essential to transition from experimental feasibility to clinical adoption.

## Figures and Tables

**Table 1:** (Referred to in Section 2) Comparative Overview of key characteristics, mechanisms of action, clinical applications, advantages, and limitations of the two major ultrasound-based therapeutic modalities SDT and FUS in Primary CNS Tumors.

Parameter	SDT	FUS
**Primary Mechanism of Action**	Activation of sonosensitizers by ultrasound to generate cytotoxic ROS	Mechanical and/or thermal effects; includes cavitation and BBB disruption
**Type of Ultrasound Used**	Low-intensity pulsed ultrasound	Low- or high-intensity focused ultrasound (LIFU or HIFU)
**Target Area**	Intracellular tumor regions containing sonosensitizers	Tumor tissue, vasculature, and BBB
**Adjunct Agents Required**	Yes—Sonosensitizers (e.g., 5-ALA, porphyrins)	Optional—Microbubbles for BBB opening
**Therapeutic Effects**	ROS-mediated apoptosis, necrosis, and immunogenic cell death	Tumor ablation, BBB modulation, enhanced drug delivery, vascular disruption
**Drug Delivery Enhancement**	Indirect (via tumor sensitization and increased ROS)	Direct (via transient BBB opening and vascular permeability)
**Immune Modulation**	Promotes immunogenic cell death and potential synergy with immunotherapies	Facilitates immune cell infiltration through microenvironmental disruption
**Clinical Stage**	Early clinical trials (e.g., recurrent glioblastoma)	Phase I/II trials for BBB opening and drug delivery; clinical use in ablation (e.g., tremor)
**Advantages**	Non-invasive; selective cytotoxicity; deeper tissue penetration than photodynamic therapy	Non-/minimally invasive; enables localized drug delivery; MRI-guided precision
**Limitations**	Requires selective uptake of sonosensitizers; ROS effects depend on tumor oxygenation	Requires optimization of sonication parameters; risk of edema or hemorrhage at high intensity
**Combination Strategies**	SDT + chemotherapy or immunotherapy for synergistic ROS and drug effects	FUS + chemotherapy, immunotherapy, or gene therapy for enhanced intratumoral delivery

**Table 2:** Clinical and Preclinical Applications of Ultrasound-Based Modalities in Primary CNS Tumors.

Study/Technology	Study Design (Phase)	Model/Population	Objective/Key Findings	Ultrasound Device	Ref.
SonoCloud-9 + Carboplatin	Clinical (Phase I/II)	Recurrent GBM patients	Repeated BBB opening to enhance chemotherapy delivery; Safe, effective BBB opening; measurable PK effects	SonoCloud-9 implantable 9-emitter device	[36]
LIPU + Paclitaxel	Clinical (Phase I)	Recurrent GBM	Enhance paclitaxel and carboplatin delivery via ultrasound; Safe; consistent BBB opening; increased intratumoral drug levels	Skull-implantable ultrasound device	[40]
Sonobiopsy	Prospective clinical (Early)	High-grade glioma patients	Increase ctDNA release by transient BBB disruption; Significant increase in ctDNA and tumor-specific mutations	Diagnostic ultrasound + microbubbles	[41] + NCT05281731 †
SDT with 5-ALA	Clinical (Phase I/II)	Recurrent GBM	Activate 5-ALA to generate ROS under ultrasound; Favorable safety; median Overall survival ~14 months	SDT-capable FUS system	[42]
Thermo-sonic ablation	Preclinical	Murine glioma	Pulsed FUS for localized ablation; Increased necrosis with minimal collateral injury	Pulsed FUS	N/R
Ultrasound-guided margin detection (FUS + hyperspectral imaging)	Experimental	Brain tumor surgery	Improve margin delineation; Better visualization of tumor boundaries	FUS + optical imaging	N/R
FUS + CyberKnife/intraoperative MRI	Experimental	Glioma patients	Improve targeting accuracy; Improved tumor targeting with tissue preservation	FUS + radiosurgery	N/R
LIBERATE Trial (LIFU + microbubbles)	Clinical (Ongoing)	GBM patients	Improve liquid biopsy sensitivity (ctDNA, cfDNA); Preliminary increase in circulating tumor DNA	MRI-guided LIFU	NCT04614283 †
FUS + Panobinostat (DIPG/DMG)	Preclinical	Orthotopic murine DIPG	Increase drug penetration; 3× increase in intratumoral concentration; tumor reduction	MRI-guided FUS	[38]
FUS + Radiotherapy (39 Gy) in DMG	Preclinical	Mouse DMG model	Assess safety of combining RT with FUS-mediated BBBO; Safe, feasible, no added toxicity	FUS	[37]
FUS-mediated immune modulation	Preclinical	Murine glioma	Assess effect of BBBO on tumor microenvironment; Increased lymphocyte infiltration; immune activation	FUS	N/R

† Ongoing trial; full results not yet published. Registry available at ClinicalTrials.gov. N/R: Not registered; experimental or preclinical study without a formal trial registry entry.

## Data Availability

No new data were created or analyzed in this study.
